# Felty syndrome: a case report

**DOI:** 10.1186/s13256-021-02802-9

**Published:** 2021-05-27

**Authors:** Anupam Gupta, Aryan Abrahimi, Aesha Patel

**Affiliations:** 1 Touro College of Osteopathic Medicine, 60 Prospect Avenue, Middletown, NY 10940 USA; 2 Orange Regional Medical Center, Middletown, NY USA

**Keywords:** Anemia, Felty syndrome, Neutropenia, Pneumonia, Rheumatoid arthritis, Splenomegaly

## Abstract

**Background:**

Felty syndrome is a rare manifestation of chronic rheumatoid arthritis in which patients develop extraarticular features of hepatosplenomegaly and neutropenia. The typical presentation of Felty syndrome is in Caucasians, females, and patients with long-standing rheumatoid arthritis of 10 or more years. This case report presents a patient with an early-onset and atypical demographic for Felty syndrome.

**Case presentation:**

Our patient is a 28-year-old African American woman with past medical history of rheumatoid arthritis diagnosed in 2017, asthma, pneumonia, anemia, and mild intellectual disability who was admitted to inpatient care with fever, chills, and right ear pain for 7 days. The patient’s mother, also her caregiver, brought the patient to the hospital after symptoms of fever and ear pain failed to improve. Our patient was diagnosed with sepsis secondary to pneumonia and urinary tract infection. She had been admitted twice in the past year, both times with a diagnosis of pneumonia. During this visit in September 2019, it was discovered that the patient had leukopenia and neutropenia. Bone marrow biopsy revealed increased immature mononuclear cells with left shift and rare mature neutrophils. During the hospital course, the patient was provisionally diagnosed with Felty syndrome and treated with adalimumab and hydroxychloroquine for her rheumatoid arthritis. Her sepsis secondary to pneumonia and urinary tract infection was treated with ceftriaxone and doxycycline, which was later switched to cefepime because of positive blood and urine cultures for *Pseudomonas aeruginosa*. She was discharged with stable vital signs and is continuing to control her rheumatoid arthritis with adalimumab.

**Conclusion:**

This case report details the clinical course of sepsis secondary to pneumonia and urinary tract infection in the setting of Felty syndrome. Our patient does not fit the conventional profile for presentation given her race, age, and the length of time following diagnosis of rheumatoid arthritis.

## Introduction

Described in 1924 by American physician Augustus Felty, “Chauffard-Still-Felty syndrome,” or simply Felty syndrome (FS), refers to a severe form of rheumatoid arthritis (RA) that often presents clinically with splenomegaly and neutropenia [1, 2]. This triad of features do not have to be complete in all patients, but neutropenia, defined as absolute neutrophil count of less than 1500/mm^3^, is a hallmark feature and must be present for diagnosis of Felty [1, 3]. Additional symptoms associated with Felty syndrome include fever, anemia, mucosal and skin ulcers, respiratory tract infections, thrombocytopenia, and lymphadenopathy [3, 4]. Felty is a rare condition that has been reported to present in 1–3% of RA patients and is more prevalent in women, often diagnosed during the fifth to seventh decade of life [1, 3, 4]. The exact cause is unknown, but it is found to have higher incidence in Caucasian populations as compared  to African American [4]. Felty is also often seen in patients having chronic active RA for 10 or more years, positive for rheumatoid factor (RF), and positive for HLA-DR4 [1, 2, 5–7]. In this report, we present an atypical case of early-onset Felty syndrome with underlying RA. Informed consent was obtained from the patient’s mother, her health proxy.

## Case presentation

A 28-year-old African American female with a 3-year history of rheumatoid arthritis presented to the emergency department (ED) in September 2019 with fever of 101.5 °F, chills, and right ear pain. This ED visit was preceded by multiple hospital admissions for similar symptoms of fever, chills, and productive cough. In July 2019, 2 months prior to the ED visit, the patient had an admitting diagnosis of sepsis secondary to bilateral community acquired pneumonia treated with doxycycline and cefuroxime, but the course was complicated by neutropenia.

One week prior to her September 2019 hospital admission, the patient went to the urgent care for persistent fever for 3 days and right ear pain. She was diagnosed with otitis media and started on amoxicillin. The patient also presented with multiple lip ulcers and was given lidocaine. The mother, her health proxy, noted the symptoms did not improve after 2 days, so she brought her to the ED. Patient was admitted for sepsis secondary to right middle lobe community acquired pneumonia and urinary tract infection. In addition to RA, the patient’s medical history includes asthma, pneumonia, mild intellectual disability, and anemia. Due to the patient’s mental disability, her mother, who is her primary caregiver and health proxy, provided the review of system. According to the mother, associated symptoms included fever, chills, fatigue, ear pain, sore throat, productive cough, abdominal pain, and myalgia. The mother denied any recent travel or any sick contacts. No nausea, vomiting, diarrhea, or dizziness were noted. There is no family history of rheumatoid arthritis, felty syndrome, or recurrent infections. There is no history of previous or current alcohol, smoking, or recreational drug use. The patient was not sexually active. Physical examination revealed the patient was febrile at 101.5 °F, tachycardic at 117 beats per minute with normal rhythm, intact distal pulses, normal breath sounds bilaterally with no wheezing, bilateral tympanic membrane injection without effusion, pharyngeal erythema, lower lip ulcers, abdominal tenderness, hepatomegaly, splenomegaly, and no active synovitis but decreased range of motion of right wrist. Pertinent laboratory tests showed white blood cell count of 3.50 × 10^3^/μL with absolute neutrophil of 25 cells/mm^3^, and platelet count of 286 × 10^3^/μL. Hemoglobin was 7.6 g/dL, hematocrit of 32.4%, and mean corpuscular volume of 71.1 fL. Peripheral blood smear showed no significant abnormalities with normal reticulocytes. Blood and urine cultures showed growth of *Pseudomonas aeruginosa*. Tests for *Streptococcus pneumoniae*, epstein–barr, herpes simplex, and influenza A and B showed negative findings. C-reactive protein was found elevated at 19.8 mg/L, and lactic acid was 2.5 mmol/L. Chest radiography in anterior posterior view showed infiltrates in the middle portion of the base of the right lung. Chest computed tomography showed no evidence of pulmonary embolism. Abdominal ultrasound showed hepatosplenomegaly with spleen measuring in the upper limits of normal. Hematology consult recommended bone marrow biopsy. The pathological evaluation of bone marrow revealed findings of normocellular focal spicules and maturing trilineage hematopoiesis, no evidence of overt dysplasia, increased immature mononuclear cells, and left shift with very rare mature neutrophils.

The provisional diagnosis was Felty syndrome. Treatment was initiated with systemic acyclovir and Abreva ointment for lip ulcers and adalimumab, hydroxychloroquine, and  azathioprine for RA. She was also supplemented with vitamin B1, B6, and B12. During the hospital stay, the patient was started on intravenous azithromycin for pneumonia and urinary tract infection (UTI). She developed shivering, elevated temperature, shortness of breath, oxygen saturation in low 70%, tachycardia, and appeared to be grunting. The patient did not lose consciousness but began shaking uncontrollably without any swelling or rash. She was diagnosed with azithromycin allergy and was given benadryl and solu-medrol intravenously. Her antibiotics were changed to intravenous ceftriaxone and doxycycline, which were changed to intravenous cefepime after positive growth of *Pseudomonas aeruginosa*. During her stay, azathioprine and hydroxychloroquine were held because bone marrow biopsy did not show any evidence of large granular lymphocytic (LGL) or lymphoproliferation. Overall the treatment was tolerated well, and the absolute neutrophil count improved. On the day of discharge, the patient was afebrile with stable vital signs and oral mucosa/lip ulcerations resolved. She currently remains on adalimumab with controlled rheumatoid arthritis.

## Discussion

Rheumatoid arthritis is a systemic autoimmune inflammatory disease characterized by synovitis with pannus formation and joint destruction [2]. RA often presents with extraarticular features such as interstitial lung disease, sjogren’s syndrome, pericarditis, osteopenia, vasculitis, episcleritis, and rheumatoid nodules [2]. Felty syndrome is a clinical diagnosis with no specific diagnostic test. FS most commonly includes a triad of long-standing RA, hepatosplenomegaly, and neutropenia. Additionally, clinical manifestations of FS include anemia, thrombocytopenia, recurrent bacterial infections, skin ulcers, idiopathic portal hypertension, and increased risk for developing hematological malignancies such as non-hodgkin’s and hodgkin’s lymphoma [1, 4, 8, 9]. This disease has a poor prognosis with increased mortality due to recurrent infections such as pneumonia and UTI complicated by sepsis. Previous studies on Felty syndrome have shown that pathogenesis of neutropenia is multifactorial. It comes as a result of underproduction of neutrophils and autoantibodies to neutrophils, which leads to antigen–antibody complexes aggregating in the spleen, causing splenomegaly [6]. However, there has been a lack of evidence on whether severity of neutropenia correlates to the degree of splenomegaly. Felty syndrome presents more commonly in women and Caucasian population, with diagnosis in the fifth or sixth decade of a patient’s life with a long-standing history of RA of 10 or more years. FS is suggested to be less prevalent in African American population because of underexpression of the HLA-DR4 gene [10, 11]. Prior research has shown HLA-DR4 to be present in over 90% of cases of Felty [7].

Felty syndrome can be misdiagnosed with any systemic condition causing lymphopenia with neutropenia. Disease processes with similar presentation include systemic lupus erythematosus (SLE), LGL leukemia, amyloidosis, sarcoidosis, and autoimmune disorders causing leukemias or lymphomas. Splenomegaly and neutropenia can be induced by drugs, or can be caused by infections due to epstein–barr virus, tuberculosis, malaria, tick-borne anaplasmosis, or human immunodeficiency virus (HIV). The physical findings and hematological manifestations of SLE and LGL make these inflammatory processes significantly higher on the differential diagnosis for Felty. SLE can be distinguished from Felty by a prominence of lymphopenia rather than neutropenia, hemolytic anemia, and presence of nephritic/nephrotic syndrome, photosensitive rash, and central nervous system disease [1, 4]. Large granular lymphocytic leukemia can be differentiated from Felty by the findings of LGL cells on peripheral smear and bone marrow biopsy [2, 4].

In this case, the patient presented with a history of recurrent pneumonia, UTI, and lip ulcers with neutropenia and lymphopenia. This presentation correlates with the traditional Felty presentation of joint disease, extraarticular hematological manifestations, recurrent respiratory infections, and physical findings of hepatomegaly, splenomegaly, and lip ulcers. What makes this case of Felty syndrome atypical is the demographics of the patient and early-onset presentation of the disease. The patient is a 28-year-old African American woman with a 3-year history of RA. Patient’s medical history places her two decades younger than the average age of presentation for FS as well as in a racial group in which FS diagnosis is relatively rare. There are numerous case presentations on Felty syndrome, but they are insufficient in regard to early onset in African American patient population.

There is no definitive treatment for Felty syndrome. The management for FS is supportive and directed towards controlling the underlying RA while also improving the neutropenia to prevent life-threatening infections. Initial management of RA with neutropenia includes low-dose methotrexate with folic acid, glucocorticoids, and recombinant granulocyte colony-stimulating factor (G-CSF) or filgrastim [12]. The role of glucocorticoids and other disease-modifying anti-rheumatic drugs (DMARDs) such as sulfasalazine, cyclophosphamide, and hydroxychloroquine is to control the inflammation, while G-CSF is a growth factor that improves the neutropenia by inducing neutrophil production [13]. Use of tumor necrosis factor (TNF)-α inhibitors such as etanercept and adalimumab have also been used to manage FS. Rituximab, as a second-line agent, has shown good efficacy for treatment resistant or refractory FS [12, 13]. Patients with Felty syndrome that has proven resistant to conventional antiinflammatory therapy may qualify for splenectomy as the last resort. Previous studies have shown an improvement in neutropenia in 60% of patients following the procedure [14]. Follow-up studies of postsplenectomy patients at 6 months reported 80% of patients had demonstrated normal levels of neutrophils [14]. This patient has been receiving long-term Adalimumab for management of her RA.

## Conclusion

Felty syndrome is a rare presentation of rheumatoid arthritis that manifests as a triad of chronic rheumatoid arthritis, neutropenia, and splenomegaly. Despite this disease being described more than 80 years ago, there is no definitive treatment and management regimen for FS. This case details the presentation and clinical course of a young African American patient with relatively recent diagnosis of RA who developed sepsis secondary to pneumonia and UTI in the setting of neutropenia. She was admitted, her infections were treated with antibiotics, and the patient was discharged in stable condition to continue controlling her RA with Adalimumab.

## Data Availability

Background information was obtained by a literature search using PubMed and Google Scholar. Keywords used for the search included “felty syndrome,” “rheumatoid arthritis,” “anemia,” “neutropenia,” “hepatomegaly,” and “splenomegaly,” which led us to 14 articles that are in the reference list. Individual patient information was derived by Orange Regional Medical Center patient chart review.
